# Safety of Safety Evaluation of Pesticides: developmental neurotoxicity of chlorpyrifos and chlorpyrifos-methyl

**DOI:** 10.1186/s12940-018-0421-y

**Published:** 2018-11-16

**Authors:** Axel Mie, Christina Rudén, Philippe Grandjean

**Affiliations:** 1 Karolinska Institutet, Department of Clinical Science and Education, Södersjukhuset, 11883 Stockholm, Sweden; 2 Swedish University of Agricultural Sciences (SLU), Centre for Organic Food and Farming (EPOK), Ultuna, Uppsala, Sweden; 3 Department of Environmental Science and Analytical Chemistry, Stockholm University, Stockholm, Sweden; 4 University of Southern Denmark, Department of Public Health, Odense, Denmark; 5 Harvard T.H. Chan School of Public Health, Department of Environmental Health, Boston, MA USA

**Keywords:** Bias, Developmental neurotoxicity, Pesticides, Toxicity testing

## Abstract

Authorization of pesticides for market release requires toxicity testing on animals, typically performed by test laboratories on contract with the pesticide producer. The latter provides the results and summary to the regulatory authorities. For the commonly used pesticide chlorpyrifos, an industry-funded toxicity study concludes that no selective effects on neurodevelopment occur even at high exposures. In contrast, the evidence from independent studies points to adverse effects of current exposures on cognitive development in children. We reviewed the industry-funded developmental neurotoxicity test data on chlorpyrifos and the related substance chlorpyrifos-methyl. We noted treatment-related changes in a brain dimension measure for chlorpyrifos at all dose levels tested, although not been reported in the original test summary. We further found issues which inappropriately decrease the ability of the studies to reveal true effects, including a dosage regimen that resulted in too low exposure of the nursing pups for chlorpyrifos and possibly for chlorpyrifos-methyl, and a failure to detect any neurobehavioral effects of lead nitrate used as positive control in the chlorpyrifos study. Our observations thus suggest that conclusions in test reports submitted by the producer may be misleading. This discrepancy affects the ability of regulatory authorities to perform a valid and safe evaluation of these pesticides. The difference between raw data and conclusions in the test reports indicates a potential existence of bias that would require regulatory attention and possible resolution.

**Electronic supplementary material:**

The online version of this article (10.1186/s12940-018-0421-y) contains supplementary material, which is available to authorized users.

## Background

Pesticide producers are responsible for the safety of their products. Due to their inherent toxic properties, market release and subsequent reauthorizations require a comprehensive and highly regulated risk assessment, although specific rules differ between countries. The toxicity testing is provided by the producer and is reviewed by regulatory authorities, such as the U.S. Environmental Protection Agency (EPA) and the European Food Safety Authority (EFSA); the latter relies on a detailed evaluation carried out by a Rapporteur Member State (RMS). Differences in conclusions on pesticide safety occur, as has recently become apparent for chlorpyrifos, a widely used organophosphate insecticide.

At the core of the toxicological risk assessment are animal tests, performed with the aim of identifying and characterizing any adverse effects, in a design in accordance with established test guidelines and under Good Laboratory Practice (GLP) requirements. Typically, a pesticide producer commissions the animal studies from external commercial laboratories, which perform the tests and generate reports under the auspices of the producer. Unfortunately, the study reports are usually not released to the public.

Independent academic studies and industry-sponsored toxicity studies may lead to fundamentally different conclusions [1], as is the case for chlorpyrifos. Thus, based on independent epidemiological*,* in vivo and in vitro studies, the evidence points to adverse health effects of chlorpyrifos exposure on the developing nervous system, associated with lowered IQ at school age, at current levels of exposure [2]. These outcomes have been observed at exposure levels far below those recognized to cause effects on brain development in an industry-funded developmental neurotoxicity (DNT) study commissioned for regulatory purposes [3, 4].

Such a discrepancy in the evidence can result in widely differing conclusions by regulatory agencies. In the U.S., a state-of-the-art EPA risk assessment recently recommended a steady-state population-adjusted dose (ssPAD) between 0.0012 and 0.002 μg/kg body weight (bw)/day, depending on age, as based on DNT observed in epidemiological studies and supported by other independent scientific evidence [5]. The most highly exposed subpopulation was children between 1 and 2 years of age, who had steady-state (21-day) dietary exposures of 0.027 (subpopulation median) and 0.242 μg/kg bw/day (99.9th percentile), thereby greatly exceeding their ssPAD of 0.0017 μg/kg bw/day [5, 6]. In the EU, the corresponding Acceptable Daily Intake (ADI) is currently set at 1 μg/kg bw/day, as based on erythrocyte acetylcholinesterase inhibition in rats observed in an industry-sponsored toxicity study. The epidemiological evidence and other independent studies indicating DNT at lower doses were assigned little weight in the EU assessment. The most highly exposed subpopulation identified in the EU is 2-to-4-year old German children, who had a chronic (long-term) dietary exposure of 0.126 μg/kg bw/day (subpopulation mean) [7], again exceeding the ssPAD.

Considering EPA’s and EFSA’s differing conclusions, we first examined published summary data [4] from the industry-funded chlorpyrifos DNT study [3]. These summary data suggest a possible effect of chlorpyrifos on the height of the cerebellum, one of nine evaluated regional brain dimensions, in exposed nursing rat pups, observed at all dosage levels for female juvenile pups. Such an effect may indicate damage to the architecture of the developing brain. Due to the intricate development schedule of the brain, such damage may have life-long consequences [2]. To understand these findings, we relied on freedom of information legislation to obtain access to the reports of the DNT studies of chlorpyrifos performed in 1997–1998 [3] and the structurally closely related compound, chlorpyrifos-methyl, from 2015 [8].

Two different test laboratories performed these guideline DNT studies, both sponsored by the same pesticide producer. In both studies, pregnant rats were exposed to different levels of the insecticide from day 6 of gestation until postnatal day (PND) 11 (chlorpyrifos) or 21 (chlorpyrifos-methyl), in accordance with test guidelines and GLP. Brain morphometrics were evaluated upon termination of dosing and in adult offspring. The pups’ neurobehavioral functions (motor activity, learning and memory, auditory startle response) were evaluated at two or four time points. Developmental landmarks as well as general toxicity in the dams and pups were also evaluated in the DNT studies.

### Brain morphological differences in the chlorpyrifos-exposed pups

The overall conclusion from the contract laboratory in the chlorpyrifos DNT study was that no effects on brain morphology and behavior were observed at dose levels 0.3 and 1 mg/kg bw/day (low and medium dose), while at 5 mg/kg bw/day (high dose), multiple effects were identified, although in the presence of maternal toxicity [3]. This conclusion was relayed by the sponsor in subsequent applications for authorization, most recently in 2015.

For high-dose pups, the test laboratory reported a significant reduction of total brain weight and the dimensions of several brain regions on PND 11 but not on PND 65 (see also Additional file 1). The test lab argued that these observed effects do not indicate DNT. Effects are instead interpreted as consequences of undernutrition secondary to maternal toxicity, which would be thought to affect all brain regions.

To support this interpretation, the test laboratory calculated that the average effect on all brain regions is similar to the effect on brain weight [3]. However, the use of averages could potentially mask effects on a specific brain region. Accordingly, the EPA has identified this analytical approach as an “inappropriate and inconclusive manipulation of the data” [9], but a correction was apparently not requested from the pesticide producer submitting the report.

**Fig. 1:**
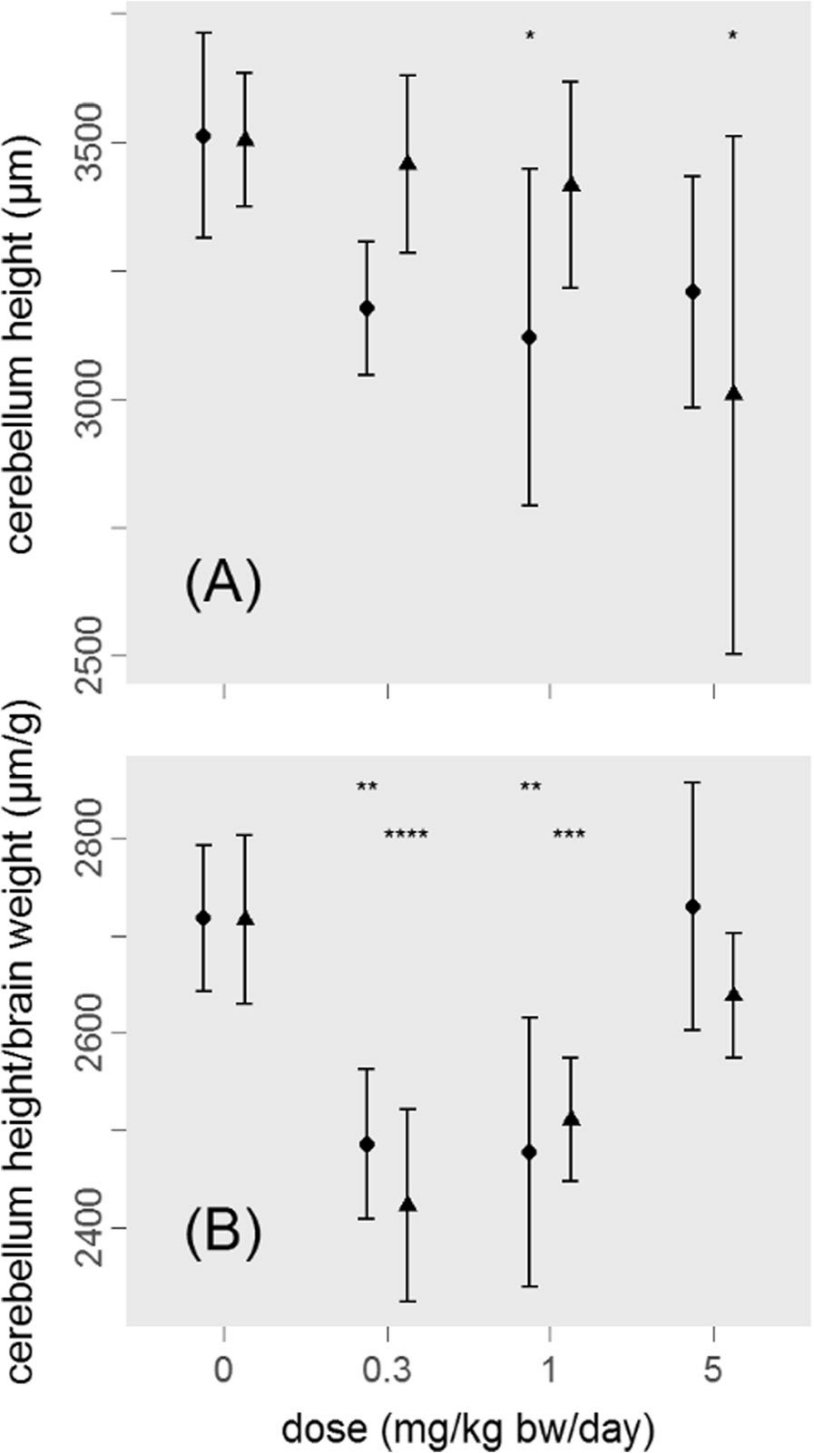
Cerebellum height in relation to chlorpyrifos exposure, PND 11 pups. 6 animals/sex/dose group. ● females, ▲males. Means ± standard deviation. (**a**) cerebellum height (data in Table 5 in [4]), (**b**) cerebellum height relative to brain weight. Asterisks denote statistical significance in Dunnett’s test: * *p* < 0.05, **p < 0.01. ***p < 0.001, ****p < 0.0001 compared to control, posthoc to significant (p < 0.05) one-way ANOVA separately for sexes

The proper way to demonstrate the absence of a sensitive target region is to express each brain regional measure relative to brain weight, which the study report also does for a limited number of cases, but not systematically [3]. Our re-analysis of the raw data shows that when expressed relative to brain weight, cerebellum height in PND 11 pups is decreased by 8–11% in low and mid-dose groups in both sexes, as compared to controls (Fig. 1). In the high dose group, absolute cerebellum height is decreased by 9–14%; however, this reduction coincides with a similarly decreased brain weight. Thus, Fig. 1a suggests a monotonic dose response relationship between dose and cerebellum height. The U-shaped dose-response relationship in Fig. 1b is consistent with an effect of chlorpyrifos on the cerebellum height at low, mid and high dose, and on overall brain weight at high dose only; the high-dose-only effect on brain weight is recognized in the study report [3]. Low- and mid-dose effects are statistically highly significant, consistent in both sexes, and observed in the absence of general maternal toxicity, hence indicating the presence of DNT at all dose levels tested. However, this analysis was not included in the DNT study report.

### Shortcomings in test design

The brain growth spurt occurs mainly postnatally in rats but prenatally in humans. Accordingly, a dosing scheme maintaining an exposure of neonatal pups equivalent to continuous in utero exposure in humans would be most relevant for addressing effects during this vulnerable developmental stage. A parallel study sponsored by the same company shows that the chlorpyrifos concentration in the blood from nursing pups decreased substantially, compared to fetal blood levels, because only a small fraction of the continued maternal exposure is transferred via milk [10]. Accordingly, the DNT study design does not appropriately model human exposure to chlorpyrifos during the full prenatal time window in humans.

We also note post-hoc changes to the statistical protocol as well as an unusually low cut-off for statistical significance (α = 0.02) in most of the study report, although a justification was not provided.

Test facilities are required to include positive control studies in DNT study reports, demonstrating their proficiency to correctly identify effects of known developmental neurotoxicants. However, in the present case, the test facility was unable to detect neurobehavioral effects of elevated developmental exposure to lead nitrate [3], although lead is a confirmed developmental neurotoxicant at very low doses [2].

The above issues limit the ability of the DNT study to detect true effects, although this was not pointed out by the test laboratory or the pesticide producer in the study report or in the submission to regulatory authorities.

### Chlorpyrifos-methyl

In the chlorpyrifos-methyl DNT study [8], brain morphology was evaluated in ten pups per sex in the control and high dose pups at PND 21 and 72. However, for cerebellum height, 40 out of 80 data points are missing in the report, with an explanation provided only for 40% of the missing data (females on PND 72, unspecified “technical error at trimming”). The test facility reported that with no other effects on the brain identified, “the absence of these few data points affecting cerebellum of females at PND 72 does not affect the interpretation of the study”.

Again, for this substance, the exposure of nursing pups may not have adequately modelled human exposure within the complete time window of prenatal vulnerability. Specifically, the study report indicates that pups were exposed via the milk while nursing [8]. However, no data on the actual exposure of nursing pups are provided.

### Implications

For the ongoing re-evaluation of chlorpyrifos in the EU, 100 unpublished industry-owned toxicology and metabolism studies have been submitted by the pesticide producer, likely totaling tens of thousands of pages. Dossiers are then examined and reviewed by authorities. Given the comprehensiveness of the submitted documentation, this procedure relies on companies submitting complete and correct information, while authorities may not have sufficient resources for an all-encompassing review.

Our review of the chlorpyrifos DNT study report identified important omissions and divergences from the guidelines and appropriate practices. For example, the raw data show an unreported deviation from the normal architecture of the developing brain at low doses; this indication of DNT would likely have spurred a request for further investigation already in 1999. The reliability of data on neurobehavioral outcomes is questioned by the failure of positive control experiments to produce positive results. Further, the study design is inappropriate for modelling human exposure during the full period of prenatal brain development in humans. A failure of both U.S. and EU authorities to act on departures from safe procedures could potentially have occurred from incomplete review of the wealth of data, perhaps coupled with exaggerated trust in the reporting from an experienced commercial laboratory. It is noteworthy that the U.S. EPA did identify several shortcomings in the chlorpyrifos DNT study, but apparently pursued only some [9, 11].

As the purpose of regulatory risk assessment of pesticides (and other industrial chemicals) is to identify and control risks to human health, the regulatory system must rely on test facilities designing and conducting the tests properly and analyzing and reporting all observed changes within test parameters.

However, in the present case, the two DNT studies seem to violate this rationale and thereby in a wider sense call into question the reliability of test results submitted, and thus also the prudence of regulatory systems relying on such tests. While conducted under the auspices of companies with a financial interest in obtaining null findings, the public health impact of not identifying existing risks may be substantial. Thus, a recent study calculated the annual costs to EU populations at €146 ($171) billion from IQ losses due to chlorpyrifos and other organophosphate exposures during pregnancy [12]. A similar calculation from the U.S. suggested annual costs of $45 billion [13].

Given the accumulating experience, several recommendations deserve renewed attention. One possibility previously considered [14, 15] is to require that future industry-funded toxicity studies be commissioned via regulatory authorities in order to avoid any perceived or real conflict of interest at the test laboratory. This solution would address concerns that industry-funded studies may downplay the risks associated with their products (e.g. [16, 17]). In addition, the test lab should be chosen by authorities based on demonstrated proficiency in addressing the required endpoints and successfully performing the tests required. Further, independent scientists should be allowed full access to all documentation from these toxicity studies, which should no longer be considered confidential business information. Finally, specifically in the present case, care must be taken not to substitute chlorpyrifos by less diligently studied organophosphate insecticides, such as chlorpyrifos-methyl, thereby potentially leading to what is known as regrettable substitution.

Low-dose effects of chlorpyrifos on the developing brain are evident both in the aggregated independent science and in the company-sponsored DNT study, but only the former have reported such conclusions. A newly proposed EPA rule [18] aims at obtaining the “best available science” and requires full public availability of all research data. A likely consequence of the proposed rule is the exclusion of important academic science from decision-making, e.g. due to ethical difficulties of publicly disclosing data from epidemiological studies [19]. However, our analysis indicates that “best available science” should require elimination of funding bias and additional reliance on all valid and relevant scientific evidence, as guided by strengths and limitations of different types of studies [20]. A narrow focus on raw data availability might augment biases of decision-making in favor of industry-sponsored studies.

Independent science and regulatory safety evaluations have different purposes and speak, to some extent, different languages. Approaches suggested e.g. by the Science in Risk Assessment and Policy (SciRAP) initiative may aid researchers in reporting and authorities in evaluating independent scientific studies for regulatory purposes [21]. Transparency and Openness Promotion (TOP) standards aim at further increasing data availability in academic science while balancing other legitimate interests such as privacy in human subjects research [19].

## Conclusions

In our review of raw data on a prominent pesticide, chlorpyrifos, and a related compound, discrepancies were discovered between the actual observations and the conclusions drawn by the test laboratory in the report submitted for authorization of the pesticide. These observations are highly relevant in view of the present legal challenges to continued chlorpyrifos use in the USA and the ongoing re-evaluation of the current approval in the EU. Although our findings may not be generalized, they suggest the existence of bias in the reporting of industry-sponsored toxicity studies. The prevalence of such (funding) bias deserves further examination. Current evaluation procedures for pesticides may need to be modified to ensure that the public is not exposed to substances that may harm human health, e.g., by commissioning required toxicity studies via regulatory authorities.

## Additional file

Additional file 1: Supplementary text (DOCX 24 kb)

## Abbreviations

ADI: Acceptable Daily Intake
bw: Body weight
DNT: Developmental neurotoxicity
EFSA: European Food Safety Authority
EPA: Environmental Protection Agency
GLP: Good Laboratory Practice
PND: Postnatal day
RMS: Rapporteur Member State
ssPAD: Steady-state population-adjusted dose
