# Supplementary material for: Safety of Safety Evaluation of Pesticides: developmental neurotoxicity of chlorpyrifos and chlorpyrifos-methyl
Source: Environ Health. 2018 Nov 16;17:77. doi: 10.1186/s12940-018-0421-y (PMC6238321; doi:10.1186/s12940-018-0421-y)
Supplement: Supplementary file 1 — Supplementary text (DOCX 24 kb) [file 12940_2018_421_MOESM1_ESM.docx]

Additional file

Supplementary text

Safety of Safety Evaluation of Pesticides:

Developmental neurotoxicity of chlorpyrifos and chlorpyrifos-methyl

Axel Mie, Christina Rudén, Philippe Grandjean.

Correspondence to: axel.mie@ki.se

Chlorpyrifos – cerebellum height and other morphometrics, PND 11 pups, summary of DNT study report

The study report on developmental neurotoxicity (DNT) [1] features two analyses of cerebellum height (and other brain morphometrics).

Initially, in the first version from May 1998, a three-way ANOVA was provided with dose Group (0 vs 5 mg/kg bw/day), Time (PND 11 + 65) and Sex as main effects and all two-factor interactions. For cerebellum height, the ANOVA results show p(Group)=0.0063, p(Group X Time)=0.0291, p(Group X Sex)=0.5407, thereby demonstrating an overall significant main effect on cerebellum height at the high dose compared to control pups ([1] p. 91).

Subsequently, in March 1999, a more detailed analysis of morphometrics data was reported, using one-way ANOVA with the factor dose Group (0, 0.3, 1, 5 mg/kg bw/day) and with Dunnett’s posthoc test [2], separately for sexes and time points. For cerebellum height on PND 11, ANOVA were significant at p=0.0295 (males) and p=0.0366 (females), with Dunnett’s test p<0.05 for high dose males and mid dose females. For eight other morphometrics, the ANOVA was significant for four (males) and one (females) outcomes on PND 11, of which one (males, high dose ant./post. cerebellum) and one (females, high dose, caudate putamen) were significant in Dunnett’s test (Table 5 in [3] and Tables 1, 3 and 4 in Supplement 2 of [1]. These statistically significant effects were dismissed by the test laboratory as either secondary to maternal toxicity at the high dose, or as inconsistent and biologically implausible [1].

Chlorpyrifos – timing and groups of brain morphometrics measurements

For chlorpyrifos, brain morphometric measurements of both sexes and all dose groups of PND 11 pup brains were performed on the same occasion. For PND 65, control and high dose brains of both sexes were analyzed on one occasion, female mid dose brains were analyzed at a later occasion, and the remaining groups were not analyzed.

Chlorpyrifos – re-analysis of cerebellum height relative to brain weight, PND 11 pups,

Guideline toxicity studies are not designed in order to advance mechanistic understanding of observed effects. Accordingly, a biological interpretation of the observed effects of chlorpyrifos on the cerebellum is not realistic within the DNT study [1] as such. In order to enable regulatory authorities to evaluate and interpret effects, and, where necessary, prescribe further testing, all findings must be reported.

Future attempts to interpret the biological significance of changes in cerebellum dimensions, for example as part of the ongoing evaluation for re-approval of chlorpyrifos in the EU, may consider the following points:

(1) Expressed in units of control group standard deviation (SD), response levels of cerebellum height relative to brain weight on PND 11 were between 2.4 and 3.4 control group SD in the low and mid dosage groups. In the absence of a specific biologically informed threshold, a response level of 1 SD may be regarded as biologically significant [4, 5], or, if the effect is considered severe, such as structural changes in the brain [4], 0.5 SD may be used as a criterion of relevance instead [5].

(2) Effects on linear measurements consistently involving one specific major brain area within a study are regarded to be a strong indicator that developmental neurotoxicity has occurred [6].

(3) Cerebellar height is determined as one of several simple brain linear morphometric measures in order to screen for effects in all neural elements within that region [6]. Such effects measures are rather coarse and non-specific and have no direct interpretation at the cellular level; changes could be a consequence of, e.g., altered cell numbers, synaptic densities, or extent of myelination.

(4) A recent human health risk assessment of chlorpyrifos, performed by the US EPA, concluded that reference values for chronic exposure should be lowered substantially, due to evidence of DNT at low exposures in epidemiological studies which is supported by other scientific evidence [7, 8].

**References**

1. Anon. **Developmental neurotoxicity study of chlorpyrifos administered orally via gavage to Crl:CD®BR VAF/Plus® presumed pregnant rats** 1998.

2. Dunnett CW: **A multiple comparison procedure for comparing several treatments with a control**. *J Am Stat Assoc* 1955, **50**(272):1096-1121.

3. Maurissen JPJ, Hoberman AM, Garman RH, Hanley TR: **Lack of Selective Developmental Neurotoxicity in Rat Pups from Dams Treated by Gavage with Chlorpyrifos**. *Toxicol Sci* 2000; **57**(2): 250-63.

4. Li AA, Sheets LP, Raffaele K, Moser V, Hofstra A, Hoberman A, Makris SL, Garman R, Bolon B, Kaufmann W: **Recommendations for harmonization of data collection and analysis of developmental neurotoxicity endpoints in regulatory guideline studies: Proceedings of workshops presented at Society of Toxicology and joint Teratology Society and Neurobehavioral Teratology Society meetings**. *Neurotoxicol teratol* 2017, **63**:24-45.

5. US Environmental Protection Agency: **Benchmark Dose Technical Guidance,** 2012. https://www.epa.gov/sites/production/files/2015-01/documents/benchmark_dose_guidance.pdf

6. Garman RH, Li AA, Kaufmann W, Auer RN, Bolon B: **Recommended methods for brain processing and quantitative analysis in rodent developmental neurotoxicity studies**. *Toxicol Pathol* 2016, **44**(1):14-42.

7. US Environmental Protection Agency: **Chlorpyrifos: Revised Human Health Risk Assessment for Registration Review. EPA-HQ-OPP-2008-0850-0195**, 2014. https://www.regulations.gov/document?D=EPA-HQ-OPP-2008-0850-0195

8. US Environmental Protection Agency: **Chlorpyrifos: Revised Human Health Risk Assessment for Registration Review. EPA-HQ-OPP-2015-0653-0454**, 2016. https://www.regulations.gov/document?D=EPA-HQ-OPP-2015-0653-0454
